# Quantification of NADPH balance during adipogenesis

**DOI:** 10.1186/2049-3002-2-S1-P40

**Published:** 2014-05-28

**Authors:** Ling Liu, Supriya Shah, Jing Fan, Kathryn Wellen, Joshua Rabinowitz

**Affiliations:** 1 Lewis-Sigler Institute for Integrative Genomics and Department of Chemistry, Princeton University, Princeton, NJ, USA; 2 Department of Cancer Biology, University of Pennsylvania Perelman School of Medicine, Philadelphia, PA, USA

## Background

NADPH provides reducing power for macromolecule synthesis and antioxidant defense. The pathways used to make NADPH under different physiological circumstances remain unclear. In growing cells, much NADPH production is coupled to nucleotide synthesis via the oxidative pentose phosphate and folate pathways. Here we examine, using isotope tracers and flux analysis, NADPH production routes in adipocytes.

## Materials and methods

Adipocytes were harvested at different time points during differentiation. To estimate NADPH demand for the reductive steps of fatty acid synthesis, lipids were isolated, saponified into fatty acids and analyzed by liquid chromatography-mass spectrometry (LC-MS). Direct measurement of NADPH deuterium labeling was applied to quantitate NADPH fluxes from the oxPPP and folate pathway [1,2]. Tracing of passage of ^13^C from glutamine into pyruvate/lactate was applied to quantitate malic enzyme flux.

## Results

Cells synthesized 30 nM/day/uL packed cell volume of acetyl units into fatty acids. To support this, a minimum of 60 nM NADPH/day/uL cell volume is needed. The pentose phosphate pathway and folate pathways contributed less than 30% of the total required NADPH. The ^13^C-glutamine tracer analysis revealed that more than 5% of pyruvate came from malate, with the associated flux, if all through NADPH-dependent malic enzyme, capable of generating 300nM NADPH/ day/uL cell volume. Thus, malic enzyme flux is potentially sufficient to produce all of the required NADPH. To confirm its role, we are working to ensure that the observed flux from malate to pyruvate is via NADPH-dependent malic enzyme as opposed to NADH-dependent malic enzyme or PEPCK-pyruvate kinase.

## Conclusion

Malic enzyme appears to be the main NADPH source in adipocytes.
